# Acetaminophen affects the duration but not the occurrence of BOLD signal decline in the dorsal hippocampus after induction of neuronal afterdischarges

**DOI:** 10.1162/IMAG.a.161

**Published:** 2025-09-22

**Authors:** Alberto Arboit, Karla Krautwald, Frank Angenstein

**Affiliations:** Functional Neuroimaging Group, Deutsches Zentrum für neurodegenerative Erkrankungen (DZNE), Magdeburg, Germany; Leibniz Institute for Neurobiology (LIN), Magdeburg, Germany; Center for Behavior and Brain Sciences (CBBS), Magdeburg, Germany; Medical Faculty, Otto von Guericke University, Magdeburg, Germany

**Keywords:** BOLD-fMRI, dentate gyrus, in vivo electrophysiology, negative BOLD response, neurovascular coupling

## Abstract

Combined in vivo electrophysiological and functional magnetic resonance imaging (fMRI) measurements were used to monitor neuronal and hemodynamic responses in the right dorsal hippocampus during and after electrical stimulation of the right perforant pathway with a short period of 20 Hz pulses. These measurements were performed under two conditions: 1.5% isoflurane (which has a long-term vasodilator effect) or 100 µg/kg medetomidine (which has a long-term vasoconstrictor effect). The stimulation elicited a short period of neuronal afterdischarges (nAD) followed by a sustained decline in fMRI BOLD signals, as previously described (Arboit et al., 2024). While the duration of nAD was similar in presence of isoflurane and medetomidine, the subsequent decline of BOLD signal was significantly longer with isoflurane than with medetomidine. However, when the same experiments were performed in the presence of acetaminophen, the duration of the sustained decline of BOLD signals became similar: acetaminophen significantly prolonged the decline in the presence of medetomidine, whereas it only slightly shortened it in the presence of isoflurane. As acetaminophen did not affect the generation and intensity of nAD, the results indicate that nAD activates at least two different neurovascular coupling (NVC) mechanisms that mediate the sustained BOLD signal decline, of which acetaminophen affects the maintenance.

## 1 Introduction

A stimulus-induced generation of ictal or short ictal-like activity in the hippocampus has been shown to cause a long-lasting decline in hippocampal blood supply, which correlates with a long-lasting decrease in BOLD-fMRI signals (Arboit et al., 2021; Farrell et al., 2020). The cellular mechanisms mediating this sustained hemodynamic response remain unknown, but are thought to play a critical role in the so-called postictal state, a clinical condition characterized by confusion, headache, nausea, and cognitive impairment. This phase typically lasts about 5–30 minutes but can also last longer than 1 or 2 days (Pottkämper et al., 2020). Therefore, shortening or even preventing this phase would improve the quality of life, especially in patients with pharmacologically resistant epilepsy. Early reports indicated that hypoperfusion during the postictal state is (at least partly) mediated by cyclo-oxygenase-1/2 (COX-1/2) mediated mechanisms and that acetaminophen, when administered before induced seizure activity, can prevent postictal hypoperfusion and corresponding hypoxia (Farrell et al., 2016). Furthermore, we could previously also demonstrate that 30 minutes after application of acetaminophen, stimulus-induced ictal-like activity did not cause a sustained decline of baseline BOLD signals as it did in the absence of acetaminophen (Arboit et al., 2021). However, this study also showed that acetaminophen alone caused a decrease in baseline BOLD signals within 10 minutes. Therefore, it remained unclear in this previous study whether acetaminophen could actually prevent the long-lasting decline in baseline BOLD signals after a brief period of ictal-like activity or if this effect was only influenced by the presence of medetomidine, a sedative commonly used in animal fMRI studies. Medetomidine is often used for fMRI studies in rodents not only because it is easy to handle but also because it has been shown to maintain stable neurovascular coupling during prolonged fMRI sessions (Sirmpilatze et al., 2019). However, it has a vasoconstrictive activity over time, thus it may interfere with hemodynamic responses elicited during ictal-like activity. To address this, we have performed similar experiments in the presence of isoflurane, an anesthetic that acts as a long-term vasodilator (Constantinides & Murphy, 2016). In a previous related study (Arboit et al., 2024), we found that in the presence of medetomidine or isoflurane, brief electrical stimulation of the perforant pathway with continuous 20 Hz pulses elicited a similar duration of ictal-like activity and a subsequently sustained decrease in hippocampal BOLD signals. Thus, if acetaminophen can effectively attenuate the ictal-activity-induced decline in basal BOLD signals (or hypoperfusion), this should be detectable in the presence of both medetomidine and isoflurane.

## 2 Material and Methods

This study was performed in parallel to an earlier study and therefore uses the same methodology as already described in detail (Arboit et al., 2024). In particular, the current study also utilizes a data set previously published in this study, namely BOLD signal changes in the right dorsal hippocampus in the absence of perforant pathway stimulation in the presence of isoflurane and medetomidine, which served as a general control for the two experimental conditions in the current studies (see Fig. 2A-top).

### 2.1 Animals

Animals were cared for and used according to a protocol approved by the Animal Experiment and Ethics Committee and in conformity with European conventions for the protection of vertebrate animals used for experimental purposes as well as institutional guidelines 86/609/CEE (November 24, 1986). The experiments were approved by the animal care committee of Saxony-Anhalt state (No. 42502-2-1406 DZNE) and performed according to the Animal Research: Reporting In Vivo Experiments (ARRIVE) guidelines. Male Wistar Han rats (age 9–13 weeks) were housed under constant temperature (23°C) and maintained on a controlled 12 h light:12 h dark cycle. Food and tap water were provided ad libitum. A total of 25 rats were included in this study (for each stimulation condition, five rats received stimulation in the presence of (I) isoflurane, (II) isoflurane and acetaminophen, (III) medetomidine, (IV) medetomidine and acetaminophen, and (V) for the two conditions without stimulation, i.e., isoflurane and acetaminophen, and medetomidine and acetaminophen, five rats were used, with at least 1 week between the two measurements). Three rats had to be excluded because no sufficient electrophysiological signal could be recorded. There was no specific assignment of the animals to the individual groups and no anonymization was done. This means that all animals were implanted with electrodes, even if not all animals were stimulated in their experiment. For the other two conditions without stimulation, that is, isoflurane (five rats) and medetomidine (eight rats), we used data that were recorded at the same time and presented in a recent publication (Arboit et al., 2024).

### 2.2 Surgical procedures

Electrode implantation was performed as previously described in detail (Angenstein et al., 2007; Arboit et al., 2021). In brief, for electrode implantation, the rats were anesthetized with pentobarbital (40 mg/kg, i.p.) and placed into a stereotactic frame. A bipolar stimulation electrode (114 µm in diameter, made from Teflon-coated tungsten wire, impedance 18–20 KΩ) was placed into the perforant pathway in the right hemisphere at the coordinates AP: -7.4, ML: 4.1 mm from bregma, and DV: 2.0 to 2.5 mm from the dural surface. A monopolar recording electrode (114 µm in diameter, made from Teflon-coated tungsten wire, impedance 18–20 KΩ) was lowered into the granular cell layer of the right dentate gyrus at the coordinates AP: -4.0 mm, ML: 2.3 mm from bregma, and DV: 2.8 to 3.2 mm from the dural surface. Monosynaptic-evoked field potentials were measured during electrode implantation to control for the correct placement, especially concerning electrode depth. Grounding and indifferent electrodes (silver wires) were set on the dura through the left side of the cranium and fixed to the skull with dental cement and plastic screws. Following surgery, the animals were housed individually and given 1 week for recovery, with *ad libitum* food and water. To alleviate possible postoperative pain, the animals were given carprofen (5 mg/kg) for 2 days. The recovery phase was normal and without problems for all animals.

### 2.3 Combined fMRI and electrophysiological measurements

**Fig. 1.**
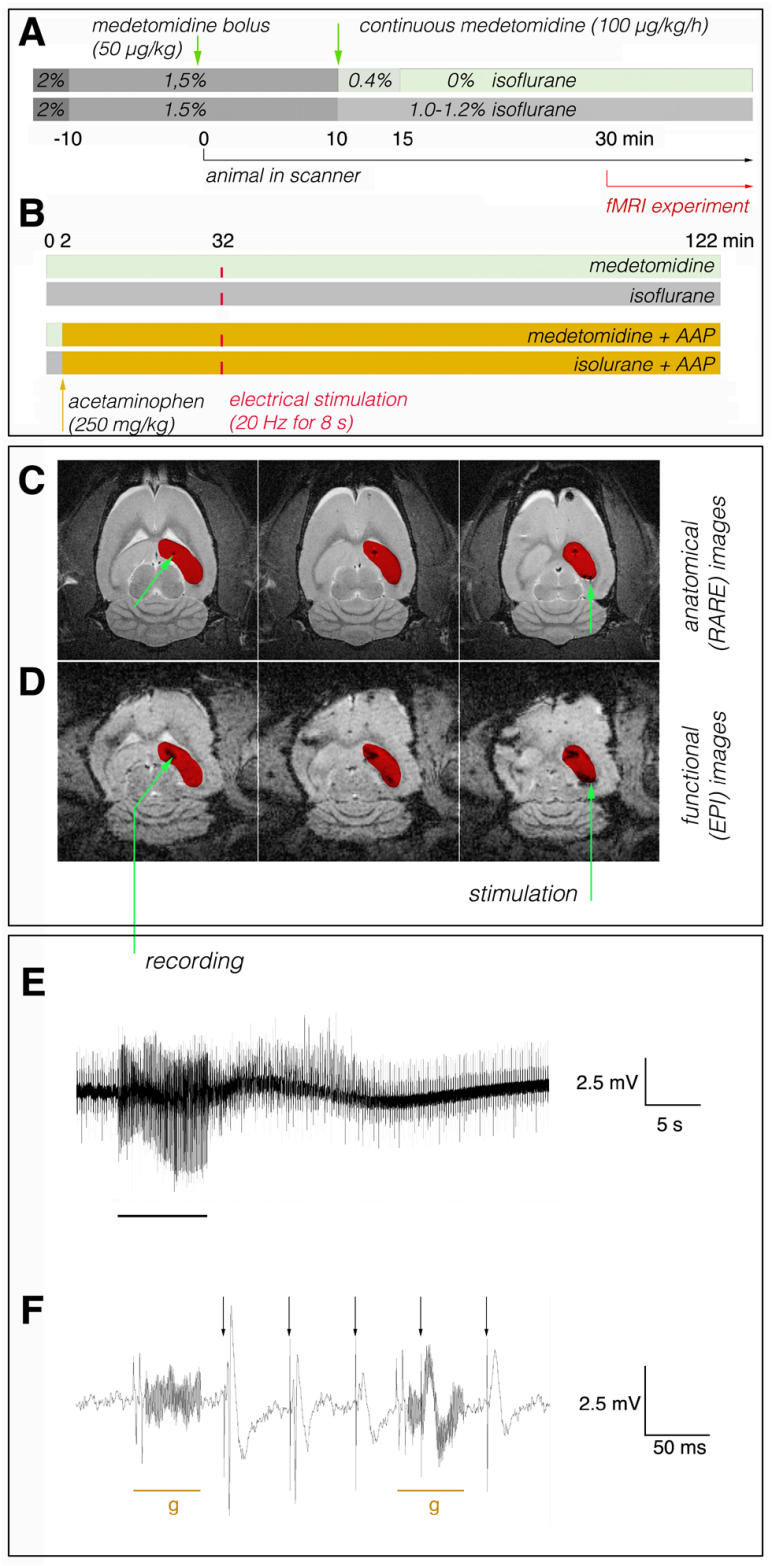
Experimental setup for combined in vivo electrophysiology and fMRI measurements. (A) Schematic view of the experimental design. Experiments were performed in the presence of medetomidine or isoflurane. (B) Schematic view of the fMRI protocol. Animals were divided into eight groups; the first four groups were measured in the absence of acetaminophen and the remaining four groups in the presence of acetaminophen. Acetaminophen was applied after 2 minutes (ochre arrow). Animals that were sedated with medetomidine were either measured without stimulation or with a stimulation after 32 minutes (indicated by the red line). Animals that were anesthetized with isoflurane were also measured either without or with one stimulation after 32 minutes. (C) Example of an anatomical image, the green arrows indicate the location of the recording electrode (left) and bipolar stimulation electrode (right). The analyzed volume of interest (VOI, right dorsal hippocampus is marked in red). (D) Corresponding functional (EPI) images with the overlaid VOI. (E) During fMRI electrophysiological signals were recorded from the right dentate gyrus. The 20 Hz stimulation period is indicated by the black bar at the bottom. (F) Detail of the raw electrophysiological recording illustrates the location and strength of the gradient artifacts (g, ochre line at the bottom) in relation to the neurophysiological responses. The black arrows indicate the stimulation artifacts.

The experimental setup for simultaneous fMRI and electrophysiological measurements during electrophysiological stimulations of the right perforant pathway in a 9.4T animal scanner was the same as recently described (Arboit et al., 2024). That means, animals were initially anesthetized with isoflurane (1.5%–2%; in 50:50 N_2_:O_2_, v:v), fixed into the head holder, and connected to recording and stimulation electrodes. Then, the anesthesia was either maintained with isoflurane (1.0%–1.2%) or switched to deep sedation by application of medetomidine (Dorbene, Pfizer GmbH, bolus: 50 µg/kg s.c. and after 10 minutes 100 µg/kg/h s.c.; Fig. 1A). The body temperature was maintained by circulating heated water in the animal cradle. The heart rate and breathing rate were monitored during the entire experiment using an MR-compatible monitoring and gating system for small animals (Model1030, SA Instruments, Inc. Stony Brooks, NY, USA); the breathing rate (under medetomidine between 40 and 60 breaths/min; under isoflurane: 50–70 breaths/min) and heart rate (under medetomidine: between 220 and 300 bpm; under isoflurane: 340–420 bpm) varied between individual animals but remained stable in all individual animals during the fMRI measurement.

Identical experiments were performed in the presence or absence of (250 mg/kg) acetaminophen, which was applied intraperitoneally 2 minutes after the start of the fMRI measurement (Fig. 1B). Thirty minutes later, the right perforant pathway was stimulated once with 160 consecutive electrical bipolar pulses (pulse width: 0.2 ms; pulse intensity: 350 µA; inter-pulse interval: 50 ms [20 Hz]). After stimulation, fMRI and electrophysiological signals were further recorded for 90 minutes; thus, the entire measurement lasted 122 minutes (Fig. 1B).

The electrophysiological responses were filtered with an antialiasing filter, that is, a low-pass filter (5000 Hz) and a high-pass filter (0.1 Hz), using an EX1 amplifier (Science Products, Hofheim, Germany) transformed by an analog-to-digital interface (power-CED, Cambridge Electronic Design, Cambridge, UK) and stored on a personal computer with a sampling rate of 5000 Hz.

All fMRI experiments were performed on a 9.4T Bruker Biospec 94/20 scanner, equipped with a BGA12 HP (440 mT/m) gradient system. An 86-mm transmit/receive volume coil (Bruker Biospin MRI GmbH, Ettlingen, Germany) was used for radio frequency (RF) excitation, and a 20-mm planar surface coil (Bruker Biospin MRI GmbH) was used for signal reception. Initially, a B0-field map was acquired, which was used for local shimming using an ellipsoid that covered the entire brain. BOLD-fMRI was performed by using a gradient-echo echo planar imaging (EPI) sequence with the following parameters: TR, 2000 ms; TE, 20.61 ms; flip angle, 90º; bandwidth, 326087 Hz; slice number, 10; slice thickness, 0.4 mm; inter slice distance, 0.1 mm; field of view (FOV), 25.6 × 25.6 mm; and matrix, 128 × 128 (in plane resolution 200 × 200 µm).

Trigger pulses generated by the scanner at the beginning of every volume—that is, every 2 second—were used to synchronize fMRI image acquisition and electrophysiological stimulation.

After fMRI measurements, 10 horizontal anatomical spin-echo-images (*T*_2_-weighted) were obtained using a rapid acquisition relaxation enhanced (RARE) sequence (Hennig et al., 1986) with the following parameters: TR, 3000 ms; TE, 33 ms; slice thickness, 0.4 mm; FOV, 25.6 × 25.6 mm; matrix, 256 × 256; RARE, factor 8; and averages, 4. The total scanning time was 6.4 minutes. The slice geometry, 10 horizontal slices, was identical to the previously obtained gradient-echo EPI.

### 2.4 VOI analysis

The functional data were loaded and converted to the BrainVoyager data format. A standard sequence of pre-processing steps implemented in the BrainVoyager QX 22.0 software (Brain Innovation, Maastricht, the Netherlands), such as slice scan time correction and 3D motion correction (trilinear interpolation and data reduction by using the first volume as a reference) were applied to each data set. We did not use any baseline correction tools, as these would mask, slowly developing or long-lasting BOLD signal changes (Supplemental Fig. S1). To illustrate this, we also performed the analysis with a baseline correction tool, i.e., a temporal filtering, high-pass filter (GLM with Fourier basis set, which includes linear trend removal, number of cycles: 3).

**Fig. 2.**
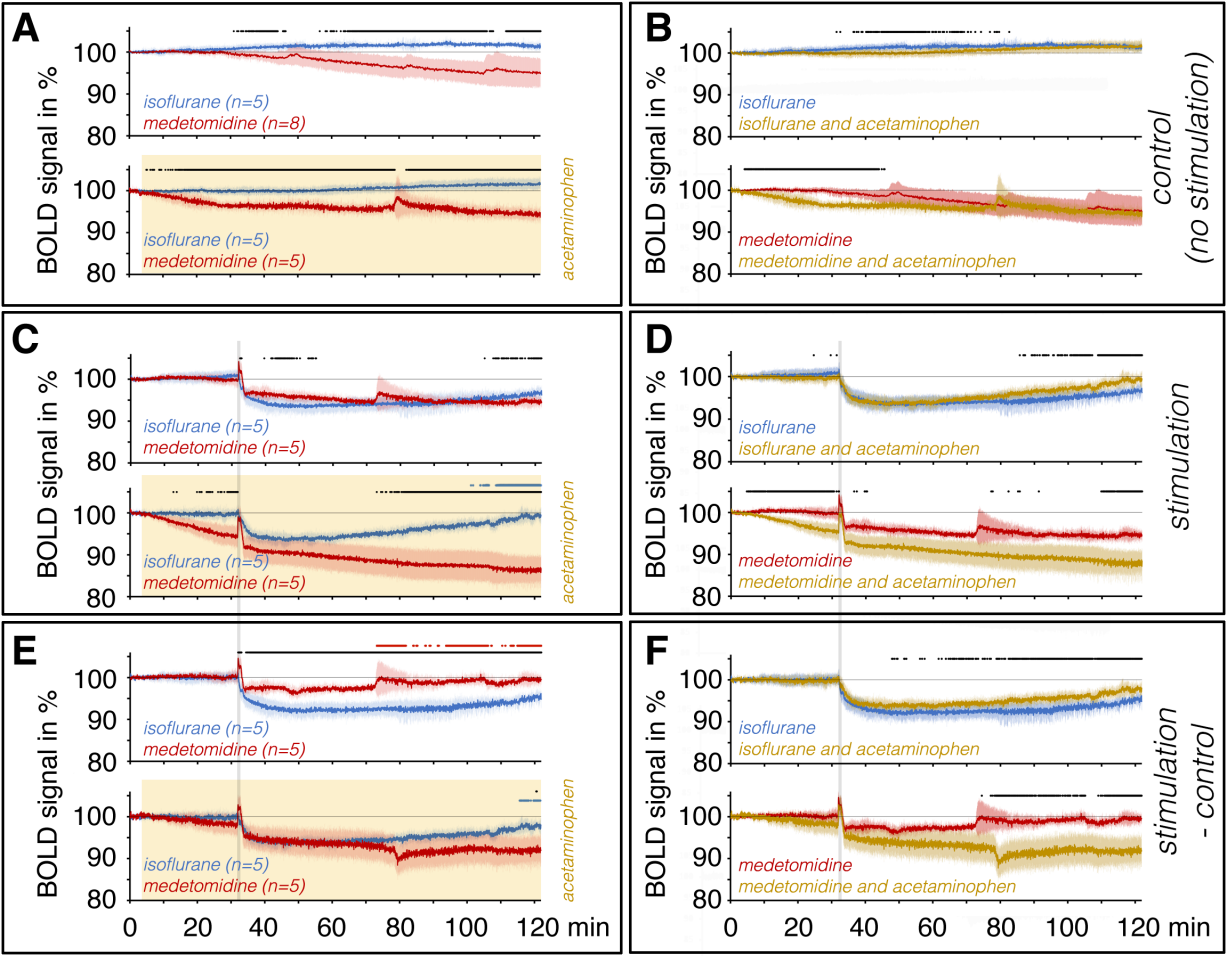
Summary of BOLD time series in the right dorsal hippocampus across all conditions. Identical experiments were performed in the presence of isoflurane (blue lines) or in the presence of medetomidine (red lines) as well as in the presence (ochre lines) or absence of acetaminophen (significant differences between the two conditions are indicated by black dots at the top of each panel). Therefore, we compared BOLD time series obtained in the presence of isoflurane or medetomidine (A, C, E) or in the presence or absence of acetaminophen (B, D, F). (A) BOLD signals in the right dorsal hippocampus developed differently during a 2-hour measurement in the presence of isoflurane and medetomidine. They increased in the presence of isoflurane, but decreased in the presence of medetomidine, leading to significant differences observed after just 30 minutes (top). When acetaminophen was applied after 2 minutes (indicated by the ochre-colored box), significant differences were already observed after 10 minutes. (B) Acetaminophen significantly delayed the increase in BOLD signal in the presence of isoflurane (top) and accelerated the decrease in BOLD signal in the presence of medetomidine (bottom). (C) An 8-second stimulation period with 20 Hz pulses caused a sustained decrease in the BOLD signal in the presence of both isoflurane and medetomidine (top). The presence of acetaminophen altered the development of BOLD signals (bottom). (D) This difference resulted from an acetaminophen-mediated earlier recovery of the BOLD signal decline in the presence of isoflurane (top) and prolongation of the BOLD signal decline in the presence of medetomidine (bottom). (E) Calculation of stimulus-related effects on BOLD signals by subtracting the intrinsic effect of isoflurane or medetomidine (see 2A top) from the traces recorded with stimulation under the same conditions (see 2C top). According to that, stimulation in presence of medetomidine caused a significantly weaker and shorter BOLD signal decline compared to the same stimulation in the presence of isoflurane (top). In the presence of acetaminophen, this difference was almost eliminated and the signal decline was significantly prolonged in the presence of medetomidine (bottom). (F) The direct comparison of acetaminophen-treated and -untreated animals revealed the effects of the drug on stimulus-mediated sustained BOLD signal decline. Thus, the BOLD signal decline recovered earlier under isoflurane, whereas it was significantly prolonged under medetomidine. Significant differences between the two BOLD time series in a graph are indicated by black dots at the top of the graph (paired t-tests, p < 0.01, Bonferroni-corrected). The period during which the BOLD signals reached 98% of the value immediately prior to stimulation (recovery) is indicated by blue dots (in the presence of isoflurane) or red dots (in the presence of medetomidine) at the top of each graph (paired t-test, p < 0.01, Bonferroni-corrected).

Each functional imaging data set was then aligned to a 3D standard rat brain by using the 3D volume tool implemented in the BrainVoyager software. The 3D standard rat brain had previously been generated from a rat of the same age and strain. The right dorsal hippocampus was marked as the volume of interest (VOI) in the 3D standard rat brain. The average BOLD time series of all voxels located in this VOI was then calculated for each animal using the VOI analysis tool implemented in the BrainVoyager software. Each BOLD time series was normalized using the average BOLD signal intensity of 100%. Normalized BOLD time series were then averaged and are depicted as mean BOLD time series ± SD (Fig. 2).

We calculated significant changes between two BOLD time series as described previously using a paired *t*-test (Arboit et al., 2024). We performed a two-sample equal variance t-test with Bonferroni correction for each time point. We considered differences to be significant when p < 0.01. Recovery of the BOLD baseline signals after stimulation was considered achieved when the BOLD signals reached 98% of the values immediately before stimulation (i.e., the average value from image 900 to 959; which corresponds to the time interval of 30-32 minutes).

Because we assumed relevant BOLD signal changes (δ) ≥ 0.5%, and baseline BOLD signal variation (σ) of 0.2%, we used a sample size of ≥ 5 animals per group (n = 16/Δ^2^; with Δ = δ/ σ). No anonymizing was done.

### 2.5 Analysis of stimulus-induced nAD recorded during fMRI

**Fig. 3.**
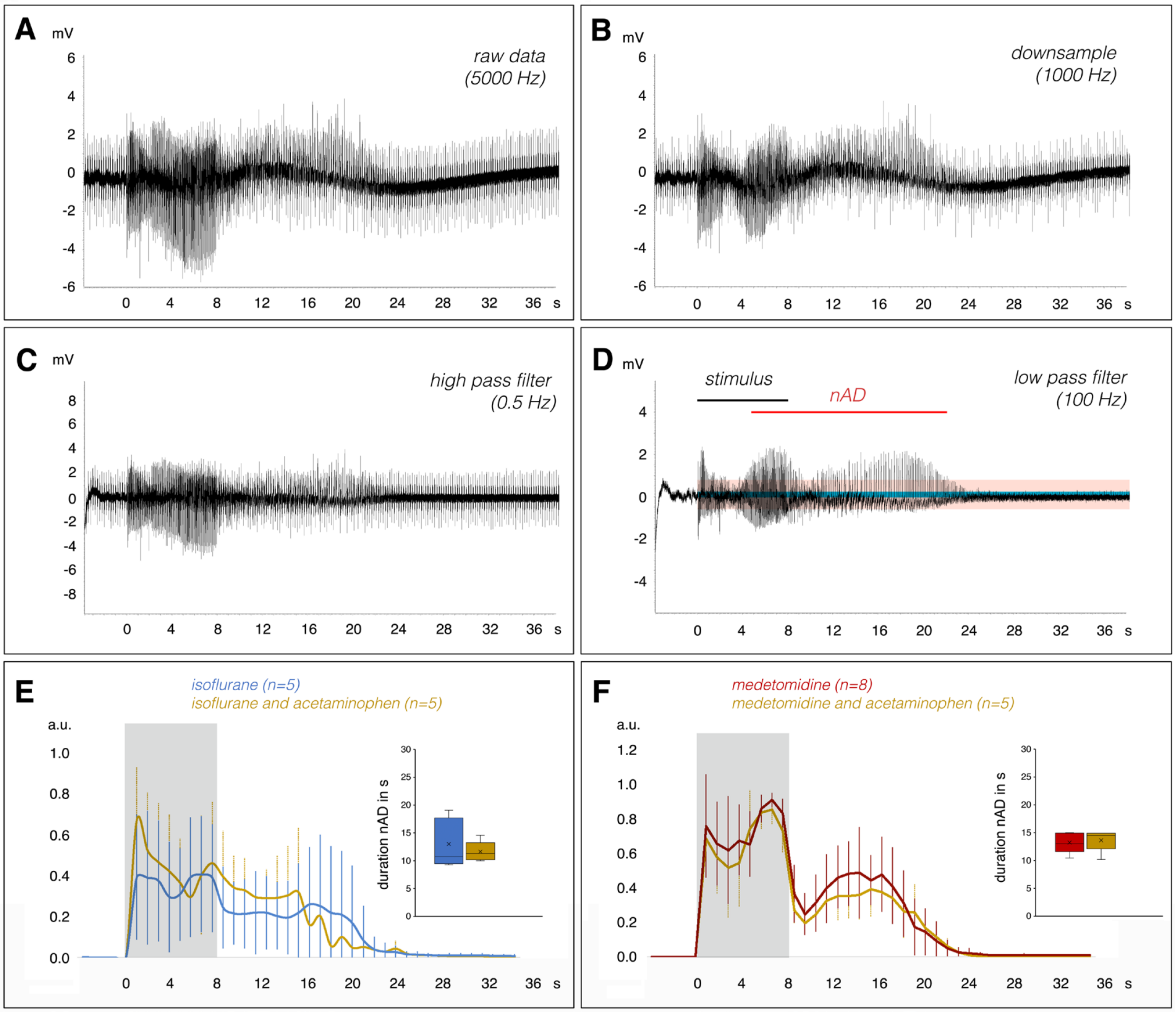
Acetaminophen did not alter the strength and duration of stimulus induced neuronal afterdischarges. (A) Electrophysiological recordings from the right dentate gyrus were (B) first downsampled, then (C) processed first by a high pass filter and finally (D) by a low pass filter (100 Hz) to minimize scanner-related artifacts. To quantify the strength and duration of nAD average ongoing neuronal activity, it was calculated (blue box) and as soon as elevated activity fell below the five-fold value (pink box) nAD were considered to end. The strength of nAD was calculated as the area under the curve for each second. (E) Average strength and duration of nAD in the presence of isoflurane (blue line) or isoflurane and acetaminophen (ochre line). No significant difference was found (a.u.: arbitrary units). Inset on the right: quantification of the nAD duration. (F) Similarly, acetaminophen did not affect the strength or duration of nAD under medetomidine (the presence of the stimulation period is indicated by the gray box).

As previously described (Arboit et al., 2024), electrical stimulation of the perforant pathway with 20 Hz for 8 second triggers nAD in the hippocampus, that is, immediately after stimulation, heavy spiking was observed (Fig. 1E). We defined the end of the nAD as the time at which the elevated activity fell below five times the average baseline amplitudes for more than 1 second. To reduce the impact of gradient artifacts on the analysis, the recordings were first downsampled to 1000 Hz and processed with a high-pass (0.5 Hz) and a low-pass (100 Hz) filter (Fig. 3A–D). The area under the curve (Fig. 3E, F) was used as a measure of intensity. To detect a significant effect of acetaminophen on nAD we used a two tailed pair t-test. Since the lack of a significant effect of acetaminophen on the duration and intensity of nAD does not necessarily prove that there is no effect (which becomes detectable at a significantly higher n number, for example), we used a two-tailed t-test (TOST) to narrow down the possible range in which a difference might still exist (Lakens, 2022).

### 2.6 Analysis of pulse-related neuronal activity recorded before and after fMRI

Spiking and postsynaptic activity of granule cells in the dentate gyrus to test pulses before and after the fMRI experiment were analyzed as described previously (Angenstein, 2019). Briefly, the amplitude of the population spike was measured in mV (from the first most positive point to the following most negative point) and the latency in ms (from the stimulus onset). All absolute measurements were averaged and are depicted as the arithmetic mean ± SD.

## 3 Results

### 3.1 Acetaminophen delayed isoflurane-induced vasodilatation and facilitated medetomidine-induced vasoconstriction

In a first set of experiments, we examined the effect of acetaminophen on the development of BOLD signals without any stimulation. As recently described (Arboit et al., 2024), in the presence of isoflurane alone, baseline BOLD signals increased in the right hippocampus to 101.62% ± 0.82% during the first hour and remained at this level until the end of the 2-hour measurement (101.39% ± 0.91%). In contrast, under medetomidine, the baseline BOLD signal in the right hippocampus remained stable for 30 minutes and then decreased over the next 70 minutes, reaching at the end of the experiment a level of 95.07% ± 3.37% (Fig. 2A-top, Supplemental Fig. S2). Thus, after approximately 30 minutes baseline BOLD signals significantly differed between the two conditions. Since the scan protocol and subsequent data analysis were identical for all measurements, the different baseline shifts could only have been caused by the effects of isoflurane and medetomidine. Under isoflurane, BOLD signals increased throughout the experiment in all regions studied, although the slope varied and was the most pronounced in the striatum. In contrast, under medetomidine, BOLD signals decreased after a certain time in the hippocampus and prefrontal cortex, while they increased in the septum and transiently in the striatum (Supplemental Fig. S3). Therefore, we assume that the BOLD baseline shifts were not only caused by general hemodynamic factors (such as blood pressure and flow), but also by an influence on neuronal activity and/or local neurovascular coupling mechanisms.

Furthermore, BOLD signals developed continuously in the presence of isoflurane, whereas in the presence of medetomidine, sudden strong BOLD signal changes occasionally occurred that were not related to obvious changes in neuronal activity. This phenomenon has been observed before and described as ‘spontaneous BOLD waves’ (Shatillo et al., 2019). Since these ‘spontaneous BOLD waves’ also occur in the prefrontal cortex but at different times (Arboit et al., 2021), they do not represent general scanner- or movement-related artifacts.

When acetaminophen was administered 2 minutes into the isoflurane condition, baseline BOLD signals initially remained at baseline levels in the right hippocampus for about 60 minutes (100.14 ± 1.14) and only then increased over the next hour to levels similar to those in the control group (right hippocampus: 101.68% ± 1.28%). This finding demonstrates that acetaminophen delayed isoflurane-mediated vasodilation (Fig. 2A-bottom, 2B-top).

Conversely, when acetaminophen was administered in the presence of medetomidine, BOLD signals in the right hippocampus began to decline immediately. After 60 minutes, these signals reached 95.99% ± 1.05%, a level untreated animals only reached after 2 hours (Fig. 2A, bottom; Fig. 2B, bottom). Thus, acetaminophen accelerated the medetomidine-mediated decrease in baseline BOLD signaling in the right hippocampus. It should also be noted that the acetaminophen concentration (250 mg/kg) used in the current and original study (Farrell et al., 2016) is far above the therapeutic concentration normally used in humans (<20 mg/kg).

Despite acetaminophen’s initial effects on the development of baseline BOLD signals, the final difference between the two experimental conditions (sedative vs sedative and acetaminophen) remained unchanged by the end of the experiment (Fig. 2B). To visualize the intrinsic effects of isoflurane or medetomidine on stimulus-related BOLD signals, control traces recorded under each type of sedation (Fig. 2A-top) were subtracted from the corresponding stimulus-related traces (Fig. 2C-top). The resulting traces are shown in Figure 2E-top. A similar approach was applied to visualize the intrinsic effects of acetaminophen, as shown in Figure 2E-bottom.

### 3.2 Acetaminophen had no effect on stimulus-evoked neuronal afterdischarges (nAD) in the hippocampus

As recently described, stimulation of the right perforant pathway with 20 Hz pulses for 8 seconds in the presence of both isoflurane and medetomidine leads to significant nAD in the hippocampus (Arboit et al., 2024). In the presence of isoflurane alone, a 20 Hz stimulation period caused nAD lasting 13.01 ± 4.42 seconds (Fig. 3A–D), whereas the same stimulation in the additional presence of acetaminophen caused nAD of 11.64 ± 1.80 seconds. In our experiments, neither the duration (p = 0.549) nor the intensity of nAD (p = 0.919) was significantly affected by the presence of acetaminophen (Fig. 3E). Based on an equivalence test, we have rejected the presence of effects on duration more extreme than -5.4 to 5.4, so we assume (with p < 0.05) that the effect of acetaminophen, if it exists, is less extreme than this range, with a 90%-confidence interval of -2,60 to 5.33 seconds. For the intensity, equivalence was shown for the delta -3.9 to 3.9, leading to a 90%-confidence interval of -3.41 to 3.83 a.u.

**Fig. 4.**
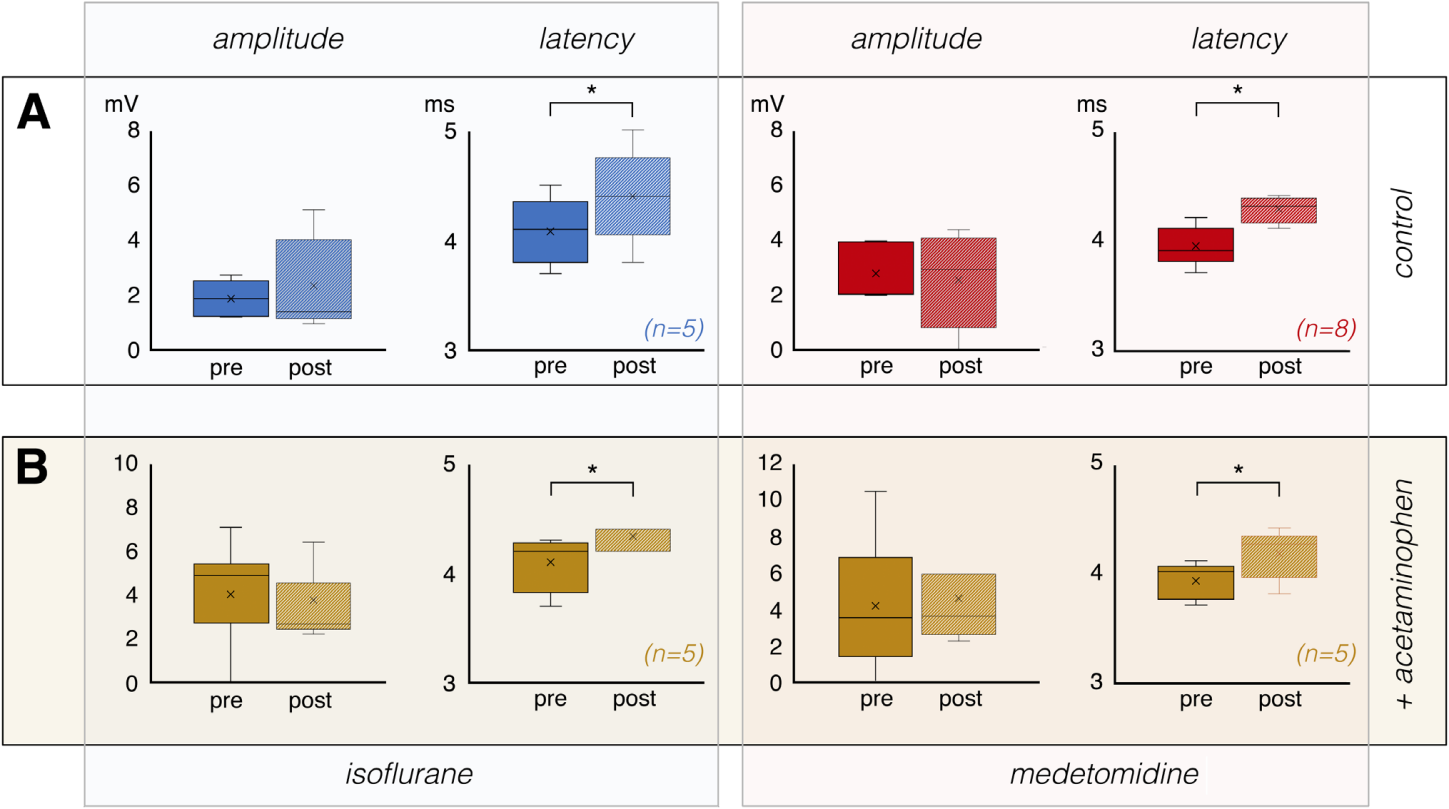
Acetaminophen does not alter the response of granular cells in the dentate gyrus to a test pulse. (A) In the presence of both isoflurane (left side) and medetomidine (right side), the 20 Hz stimulation period led to a significant increase in population spike latency (indicated by an asterisk; paired t-test, p < 0.05) that was not accompanied by a change in amplitude. Summarized are the responses to a test pulse recorded immediately before (pre) and immediately after (post) the fMRI experiment. (B) When acetaminophen was present during the 20 Hz stimulation period, the neuronal responses to the test impulses developed in the same way as under control conditions, i.e., without acetaminophen.

In the presence of medetomidine alone, 20 Hz stimulation caused nAD with a duration of 13.25 ± 1.85 seconds. When acetaminophen was included, nAD lasted 13.66 ± 1.87 seconds, again showing no significant difference (p = 0.726). According the equivalence test, we have rejected the presence of effects on duration more extreme than -2.5 to 2.5, so we assume (with p < 0.05) that the effect of acetaminophen, if it exists, is less extreme than this range, with a 90%-confidence interval of -2,48 to 1.66 seconds. For intensity (control: 10.96 ± 2.90 a.u.; acetaminophen: 9.65 ± 1.86 a.u) equivalence shown for margins of -4,2 to 4.2, leading to a a 90%-confidence interval of -1.56 to 4.17 a.u. Thus again, we detected no change in the general pattern and intensity of nAD when acetaminophen was administered 30 minutes before stimulation (Fig. 3F). In addition, acetaminophen did not modify neuronal responses to test pulses that were applied immediately before and after the fMRI measurement. Under control conditions, that is, in the presence of only isoflurane or medetomidine, the amplitude of the pulse-induced population spikes remained the same during the second measurement, while the latency increased significantly. This long-lasting effect (over 90 minutes) on perforant pathway-granule cell synapse efficacy was observed with 20 Hz stimulation under both isoflurane and medetomidine. (Fig. 4A). The same change in neuronal responses, that is, unchanged population spike amplitude and significantly increased their latency, was observed when acetaminophen was applied before the 20 Hz stimulation (Fig. 4B).

In summary, we detected no significant effect of acetaminophen on the duration, intensity or pattern of nAD triggered by 20 Hz stimulation in the hippocampus, regardless of whether isoflurane or medetomidine was present.

### 3.3 Acetaminophen shortened stimulus-induced baseline BOLD decline in the presence of isoflurane but prolonged it in the presence of medetomidine

As recently described (Arboit et al., 2024), in the presence of isoflurane, a short period of 20 Hz pulses caused no immediate transient positive BOLD response, but only a long-lasting decline of BOLD signals in the right hippocampus that lasted for more than 90 minutes (Fig. 2C-top), that is, it did not recover during the experiment. If acetaminophen was administered 30 minutes before stimulation under isoflurane, the perforant pathway stimulation also did not trigger a temporary positive BOLD response, signals in the right hippocampus, similar to what was observed in its absence. However, the decline in BOLD signals was shorter and already recovered 70 minutes after stimulation (Fig. 2C-bottom, 2D-top). Considering the effect of acetaminophen alone on the development of baseline BOLD signals (Fig. 2B top), the first significant effect of acetaminophen on the stimulus-induced decrease in BOLD signals in the right hippocampus occurred after approximately 20 minutes leading to a recovery of BOLD signals after 85 minutes (Fig. 2F-top). This indicates that, in the presence of isoflurane, acetaminophen significantly shortened the nAD-induced long-lasting BOLD signal decline in the right hippocampus, but did not modify the strength of the initial decline.

When the same stimulation was applied in the presence of medetomidine, it first caused a transient positive BOLD response that was also followed by a long-lasting decline of baseline BOLD signals in the right hippocampus that did not recover during the entire measurement (Fig. 2C-top, 2D-bottom). However, considering the intrinsic effect of medetomidine on BOLD signals during the experiment, the stimulus-dependent decline recovered after 41 minutes, thus it differed significantly from the effect observed in the presence of isoflurane (Fig. 2E-top). When the same stimulation was given 30 minutes after acetaminophen administration, the duration of stimulus-dependent decline in BOLD signals was significantly prolonged (Fig. 2C-bottom, 2D bottom), which became more obvious when the intrinsic effect of acetaminophen on baseline BOLD signals was taken into account (Fig. 2F-bottom). Again, acetaminophen only affected the duration of the sustained BOLD signal decline but did not modify the strength of the initial decline (Fig. 2F-bottom, Supplemental Fig. S4). That is, although BOLD signals decreased in the presence of medetomidine and acetaminophen before stimulation, the same decrease in BOLD signals occurred after stimulation as under control conditions. This finding suggests that the effects of acetaminophen and medetomidine are independent, resulting in an additive effect.

In conclusion, in the absence of acetaminophen, identical stimulation of the perforant pathway caused significantly different development of BOLD signals under isoflurane and medetomidine (Fig. 2E-top). However, in the presence of acetaminophen, the development of BOLD signals became similar, except for the initial transient positive BOLD response (Fig. 2E-bottom).

## 4 Discussion

In the present study, we investigated the effect of acetaminophen on the nAD-induced long-lasting decline in BOLD signals, after it was recently shown to effectively attenuate vasoconstriction in the hippocampus after seizure-like activity (Farrell et al., 2016). The main findings of the current study are: (1) acetaminophen transiently affects isoflurane- and medetomidine-mediated effects on baseline BOLD signals in the hippocampus; (2) acetaminophen does not affect the generation, duration, and severity of stimulus-evoked nAD in the hippocampus under these two conditions; and (3) acetaminophen had opposite effects on the duration of the nAD-dependent sustained decline of BOLD signals, shortening it in the presence of isoflurane but prolonging it in the presence of medetomidine.

In general, sustained changes in BOLD signals could be directly caused by a long-lasting vascular response (e.g., vasoconstriction or vasodilation) or indirectly by long-lasting changes in neuronal activity (i.e., an increase or decrease). A vascular response itself may be either directly caused by a substance (e.g., via targeting endothelial receptors and/or downstream intracellular signaling mechanisms in the endothelial cell leading to vasoconstriction or dilation) or indirectly mediated by influencing neurovascular coupling mechanisms (i.e., by affecting the release/or activation of vasoactive substances from activated neurons/glial cells). To better distinguish between these different mechanisms, we combined BOLD fMRI with in vivo electrophysiological recordings to simultaneously monitor changes in neuronal activity and hemodynamic during and after stimulation.

### 4.1 Acetaminophen and cerebral blood flow in the hippocampus

In our first experiments without electrical stimulation, acetaminophen only accelerated the medetomidine-related slowly decline of BOLD signals, as seen previously (Arboit et al., 2021), but did not enhance it (Fig. 2B, lower panel). Also, it only transiently delayed the isoflurane-related increase in BOLD signals, but did not cause a decline below the initial value. Thus, acetaminophen seemed to transiently reduce blood flow in the dorsal hippocampus, suggesting a potential (short-term) vasoconstrictive effect. However, based on our results, we can neither confirm nor deny this assumption, as this would require an experiment conducted without a sedative or anesthetic. Unfortunately, such an experiment is not currently feasible due to the long duration of the measurement.

However, under isoflurane and medetomidine, the effect of acetaminophen occurred at different time periods and with different strength. Thus, without acetaminophen, the BOLD time series under these two conditions did not differ until after 30 minutes. In contrast, following the application of acetaminophen, the BOLD time series differed as early as 10 minutes (Fig. 2A, B and Supplemental Fig. S1).

This indicates that, depending on the experimental condition, in the presence of isoflurane or medetomidine, acetaminophen differently modified the hemodynamic, as measured by the parameter BOLD signal. If acetaminophen directly influences blood flow, one would expect a similar onset and strength of action under both conditions. Since this was not the case, a direct effect of acetaminophen on cerebral blood flow is rather unlikely. Acetaminophen therefore rather interferes with the neurovascular coupling mechanisms that are specifically modulated by isoflurane and medetomidine. This fits with the fact, that so far, acetaminophen itself has not been reported to have a vasoconstrictive effect; the metabolite AM404 (at a concentration of 10 mg/kg) has even been shown to cause a transient increase in cerebrovascular blood flow (Iring et al., 2013).

### 4.2 Acetaminophen and neuronal activity

During fMRI, we recorded neuronal activity in the dentate gyrus elicited by stimulation of the perforant path with single electrical pulses, and additionally the neuronal activity that persisted after the administration of 8 seconds long 20 Hz pulse trains, the nAD. The pulse-induced neuronal activity represents synchronized spiking of the principal cells (granule cells) of the dentate gyrus, which were monosynaptically activated by glutamatergic perforant pathway fibers. This activity appears as a population spike and its amplitude (relates to the amount of synchronized spiking of the granule cells) and latency (relates to the excitability of the granule cells) can be quantified. In contrast, nAD represents neuronal activity that is likely generated in different regions of the hippocampus (hippocampus proper and dentate gyrus) and whose duration we quantified as a measure of severity.

Acetaminophen is metabolized in the brain to AM404, which in turn is an activator of cannabinoid CB1 and CB2 receptors as well as TRPV1 channels (Ohashi & Kohno, 2020). It was previously reported that activation of cannabinoid CB1 and CB2 receptors had antiepileptogenic effects, whereas their inhibition had pro-epileptogenic effects (Cristino et al., 2020), and similarly, activation of TRPV1 channels has also been shown to have antiepileptogenic properties. Consistent with these observations, high concentrations of acetaminophen were found to be anticonvulsant in certain seizure models in mice (Suemaru, Yoshikawa, Aso, et al., 2018; Suemaru, Yoshikawa, Tanaka, et al., 2018). Our data indicate that the high concentration of acetaminophen used in this study (i.e., 250 mg/kg) did not significantly modify the duration or intensity of stimulus-induced nAD under isoflurane or medetomidine (Fig. 3E, F). It is, therefore, very unlikely that the changes in BOLD signals we observed after nAD in the presence of acetaminophen were due to altered neuronal activity during the nAD. Moreover, the neuronal responses to an identical stimulus before and 90 minutes after acetaminophen administration were similar (Fig. 4A, B), suggesting that acetaminophen did not significantly alter the general excitability of granule cells in the dentate gyrus.

Nevertheless, acetaminophen affected the duration of BOLD signal decline after nAD. This suggests that, while acetaminophen did not alter the characteristics of the evoked neuronal afterdischarges, we cannot exclude the possibility that it modulates ongoing neuronal activity or other components of neurovascular coupling, as electrophysiological recordings were limited to the stimulation and immediate post-stimulation periods.

### 4.3 Acetaminophen and long-lasting NVC mechanisms

Acetaminophen was thought to attenuate postictal hypoperfusion/hypoxia by inhibiting COX-2 (Farrell et al., 2016). As described previously, COX-2 mRNA expression in hippocampal neurons increases within 30 minutes after a seizure, then peaks after 1–2 hours and remains elevated for up to 8 hours (Yamagata et al., 1993). Furthermore, the same study also revealed that normal ongoing neuronal activity maintains a low baseline expression of COX-2 mRNA, which is lost after treatment with MK801 or tetrodotoxin (TTX). Although the main products of COX-2 activity, PGE2 and PGI2, are effective vasodilators (Lacroix et al., 2015; Lecrux et al., 2011; Niwa et al., 2000), seizure- and seizure-like activity still leads to vasoconstriction. This suggests that the observed postictal state is not driven by the obvious pathway of COX-2-mediated PGE2/PGI2 formation but rather by an alternative, concomitantly triggered mechanism (not yet described). This idea is supported by the finding that genetic knockdown of COX-2 function also completely prevents postictal hypoxia (hypoperfusion) but not seizure activity (Farrell et al., 2016), indicating that COX-2 activity is absolutely required to cause vasoconstriction after a seizure.

In this study, acetaminophen alone already had an immediate effect on baseline BOLD signals in the hippocampus in the absence of stimulation (but in the presence of ongoing neuronal activity), both under isoflurane and medetomidine. As mentioned above, it is unlikely that acetaminophen alone causes vasoconstriction; instead, it may interfere with NVC mechanisms controlled by medetomidine/isoflurane.

This could fit with the previous finding that normal ongoing neuronal activity maintains a certain expression of COX-2 (Yamagata et al., 1993), which, in turn, also maintains a general vasodilation. This would be similar to the COX-1 activity, which is constitutively expressed (in astrocytes) and maintains a constant vasodilation of local brain blood vessels (Rosenegger et al., 2015).

However, when nAD caused a sustained decrease in BOLD signals, acetaminophen affected the duration of this decline differently under isoflurane and medetomidine. As recently described (Arboit et al., 2024), an 8 seconds stimulation period with 20 Hz pulses caused nADs with similar durations, but different duration and extent of BOLD signal declines under isoflurane and medetomidine. Furthermore, under medetomidine, the stimulation and the period of nAD caused a transient positive BOLD response, which was almost absent when the stimulation occurred in the presence of isoflurane. In the current study, where the stimulation occurred 32 minutes after the start of the fMRI measurement instead of 2 minutes as in the previous study, this effect was also observed. Based on these results, we concluded in the previous study that isoflurane and medetomidine affect different NVC mechanisms simultaneously activated during stimulation and nAD. Specifically, isoflurane inhibits NVC mechanisms responsible for the initial transient positive BOLD response. In contrast, medetomidine allows this transient positive response. The differing extent of the subsequent BOLD decline appears to result from the differing starting levels of the BOLD signal. With medetomidine, the transient positive response elevates the baseline, leading to a higher starting point for the decline. Conversely, with isoflurane, the absence of a clear transient positive response results in a lower starting point for the decline. Our current results indicate that acetaminophen does not influence the initial transient positive BOLD response or the subsequent, initial decline of BOLD signals after nAD. However, it does alter the maintenance phase of the decline. Therefore, we conclude that acetaminophen mainly affects NVC mechanisms that control the maintenance of the nAD-related BOLD signal decline. Since acetaminophen does not affect the initial, but only the sustained decline of BOLD signals, it implies that nAD activates distinct NVC mechanisms in parallel to control the subsequent long-lasting decline of BOLD signals.

**Fig. 5.**
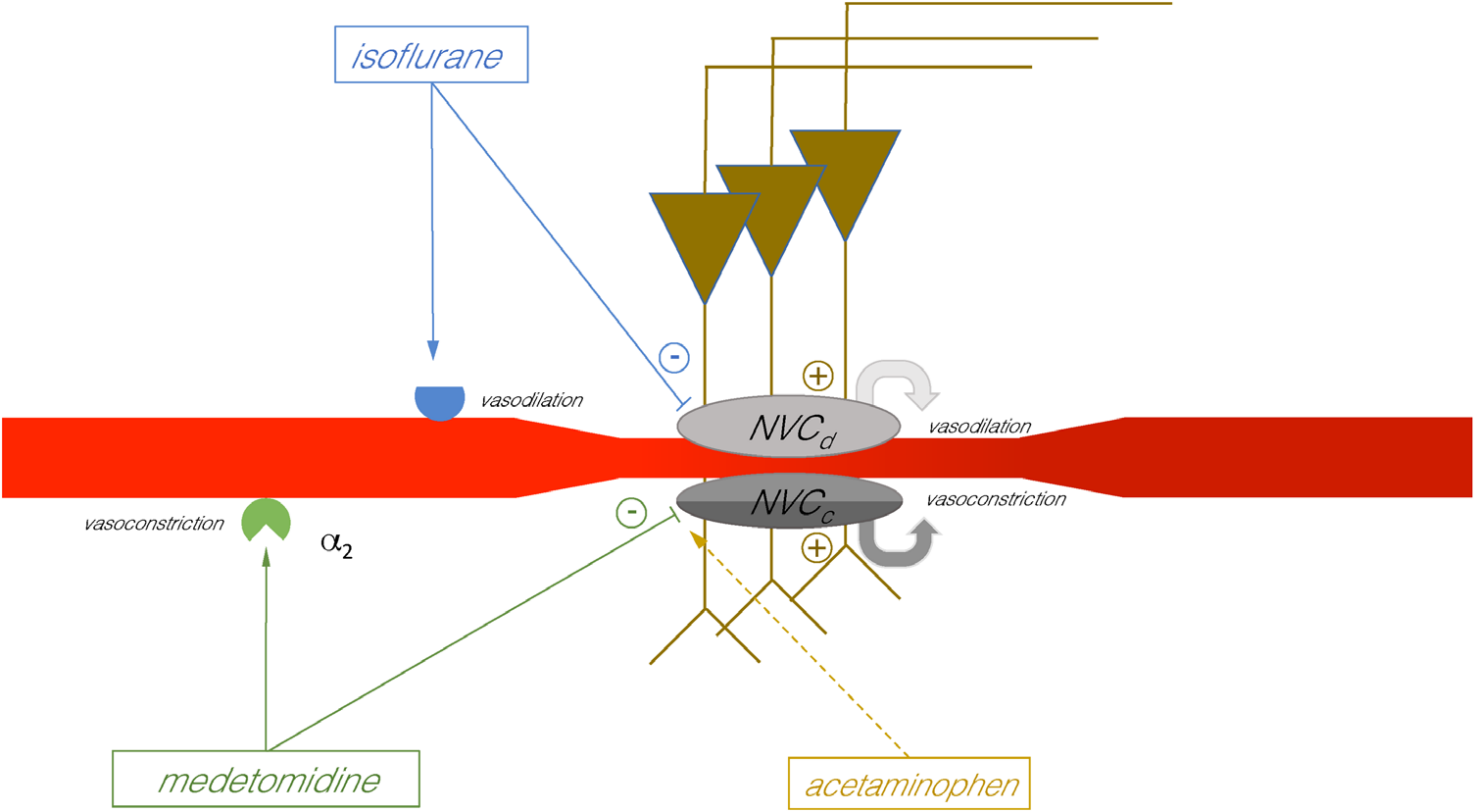
Summary of the interaction of several neurovascular coupling mechanisms (NVC). activated by a train of 20 Hz pulses. This stimulation elicited both pulse-related neuronal responses and nAD, which in turn activated NVCs that mediated both vasodilation (or functional hyperemia, detectable as a transient positive BOLD response) and vasoconstriction (detectable as a sustained decrease in BOLD signals). The presence of isoflurane during stimulation inhibits the NVC mechanism(s) that mediate vasodilation (indicated as *NVC_d_*), therefore no clear transient positive BOLD response was observed, although a sustained BOLD signal decrease was still induced (see also Arboit et al., 2024). The presence of acetaminophen does not interfere with the NVC that mediates the transient vasodilation. In the presence of both isoflurane and medetomidine, stimulation caused a long-lasting decline in baseline BOLD signals (via *NVC_c_*). The decline lasted at least 90 minutes in the presence of isoflurane and was only slightly affected by acetaminophen. In the presence of medetomidine, however, it only lasts for about 40 minutes. However, when acetaminophen was additionally administered, the decline was prolonged to a similar period of time as in the presence of isoflurane alone. Since acetaminophen did not prolong the decline in the presence of isoflurane, we hypothesize that at least two different NVC mechanisms mediate the long-lasting decline. Only the NVC mechanism, which mediates the late phase (indicated by the darker color), is inhibited by medetomidine. The inhibitory effect of medetomidine on this mechanism appears to be abolished by acetaminophen. Therefore, we hypothesize that at least two independently acting NVC mechanisms mediate the long-lasting decline of baseline BOLD signals after nAD in the dorsal hippocampus.

In the presence of medetomidine, this mechanism appears absent or inhibited. However, the mechanism is active or becomes activated in the presence of isoflurane. This would explain the two different BOLD time series in Figure 2E (upper graph), in which the BOLD signals returned to baseline within the first 40 minutes after stimulation in the presence of medetomidine. In contrast, in the presence of isoflurane, this only occurred after more than 90 minutes. Interestingly, in the presence of acetaminophen, the BOLD time series became similar except for the initial transient BOLD response, which was seen only in the presence of medetomidine. It appears that acetaminophen activated a mechanism that maintains the decline of BOLD signals only in the presence of medetomidine (Fig. 2F, lower graph, Fig. 5). Meanwhile, in the presence of isoflurane, it competes with an endogenous activated mechanism, resulting in the observed slight inhibition (Fig. 2F, upper graph). Thus, acetaminophen acts like a partial agonist for a mechanism controlling a long-lasting BOLD signal decline.

In addition, in the presence of medetomidine, acetaminophen caused a decline in baseline BOLD signals (Fig. 2B-bottom). Despite this baseline reduction, stimulus-induced nAD caused a similar decrease in BOLD signal as it did in the absence of acetaminophen (Fig. 2D bottom, Supplemental Fig. S4), suggesting that these two BOLD signal changes were caused by two different mechanisms and/or at different sites.

### 4.4 Limitations

The study was designed to monitor putative changes in BOLD signal intensities caused by the presence of acetaminophen. It turned out that the stimulus-induced changes in neuronal activity (especially the nAD) were more variable than the corresponding BOLD signal changes. Therefore, the statistical significance describing the effect of acetaminophen on nAD is lower than the effect on BOLD signals. Future studies with higher animal number can address this limitation.

While acetaminophen was injected intraperitoneally, the control animals did not receive an intraperitoneal injection, for example, saline, so that it cannot be ruled out that injection-related artifacts were present. However, since acetaminophen affected the maintenance of the nAD-mediated decrease in BOLD signal, that is, had an effect more than 60 minutes after application, this should be negligible.

## 5 Conclusions

Overall, our current results do not support the original hypothesis that acetaminophen attenuates an nAD-mediated, long-lasting decline in BOLD signals, indicative of sustained hypoperfusion. This is in contrast with an earlier study showing that acetaminophen effectively attenuates ictal activity-induced hypoperfusion (Farrell et al., 2016). However, it should be noted that this earlier study measured hippocampal blood flow using an implantable laser doppler flowmetry (LDF) probe and oxygen levels (pO2) using an optode in awake rats. Thus, no confounding effect from medetomidine or isoflurane was present in this study. Furthermore, longer lasting ictal activity was induced by a 2 minutes long period of continuous 3 Hz pulses with an intensity of 1 mA. In contrast, in the current study only short nAD were induced by an 8 seconds long period of continuous 20 Hz pulses with an intensity of 350 µA. Thus, both ictal and nAD-induced activity cause sustained hypoperfusion, but the same mechanisms do not necessarily mediate them. Either way, the current study suggests that the maintenance of sustained BOLD signaling decline due to nAD is mediated by at least two sequential neurovascular coupling mechanisms. While medetomidine seems to attenuate the later component in an acetaminophen-sensitive manner, isoflurane does not appear to affect either component. These findings extend previous observations by demonstrating that different NVC mechanisms activated by a neuronal process (e.g., nAD) can act not only simultaneously, as reported in earlier studies (Arboit et al., 2024), but also sequentially.

## Supplementary Material

Supplementary Material

## Data Availability

All data and code used in this manuscript will be available upon reasonable request from the corresponding author.
