# Supplementary material for: Acetaminophen affects the duration but not the occurrence of BOLD signal decline in the dorsal hippocampus after induction of neuronal afterdischarges
Source: Imaging Neurosci (Camb). 2025 Sep 22;3:IMAG.a.161. doi: 10.1162/IMAG.a.161 (PMC12455054; doi:10.1162/IMAG.a.161)
Supplement: Supplementary Material [file IMAG.a.161_supp.pdf]

Supplemental figures

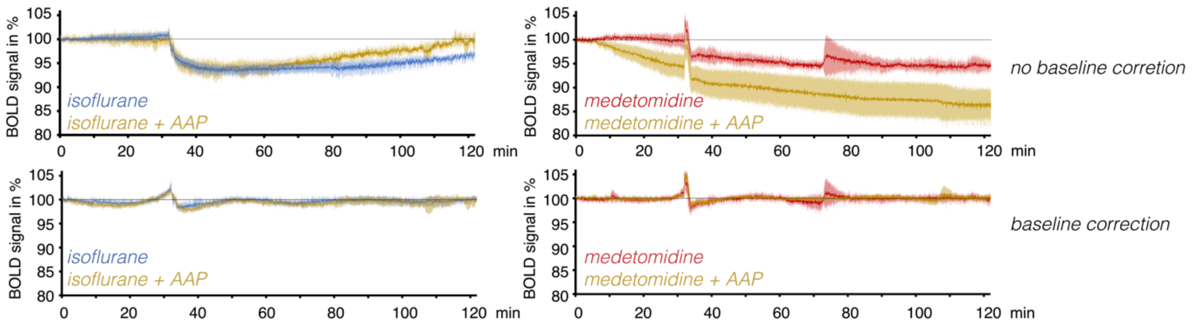

Figure S1.

Baseline correction tools mask long-term changes in stimulus-induced BOLD signaling changes, and thus the effect of acetaminophen on these changes. (**A**) In the presence of isoflurane, acetaminophen (AAP) shortened the stimulus-induced BOLD signal decline (top). When the analysis was performed with a baseline correction tool, the sustained decline of BOLD signal and the effect of acetaminophen was not detectable (bottom). (**B**) When the same experiment was performed under medetomidine, the acetaminophen-mediated, slow-developing decline in BOLD signals before stimulation and the other longer-lasting changes were not detectable. In contrast, the short-term immediate positive BOLD response was clearly detectable.

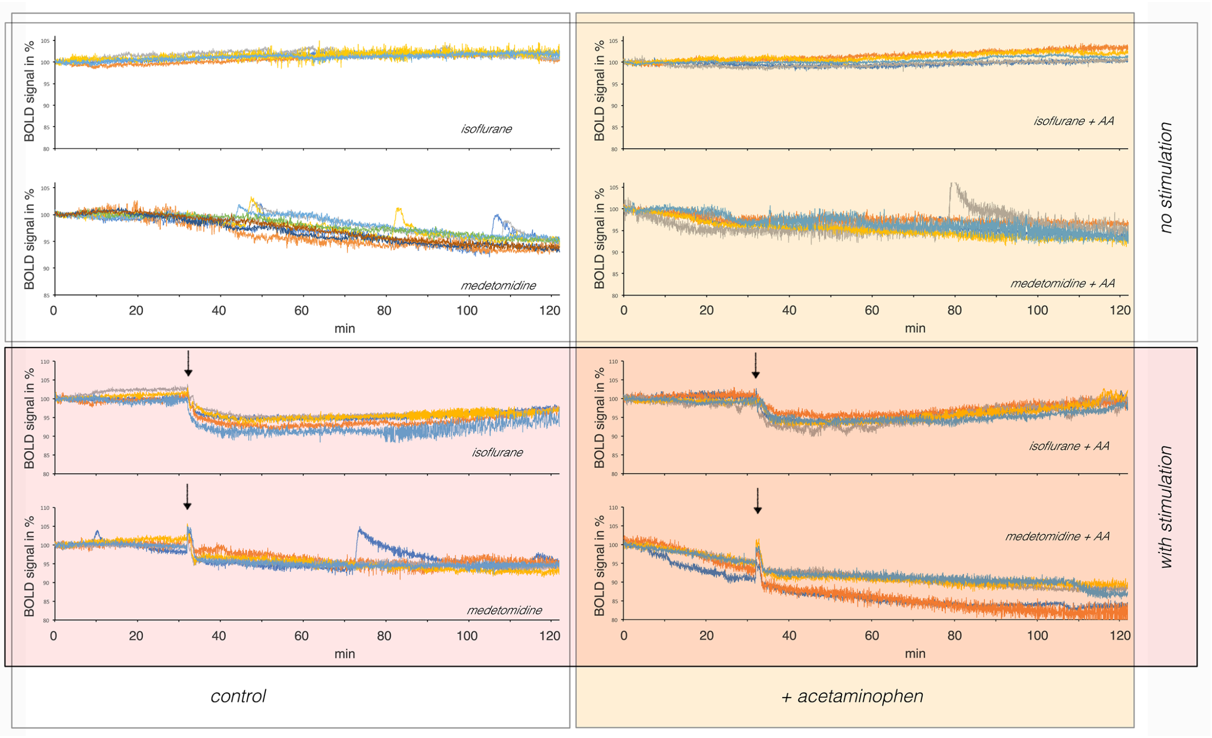

Figure S2.

Summary of all individually measured BOLD time series in the right dorsal hippocampus, measured in the presence of isoflurane or medetomidine alone (left side) or in combination with acetaminophen (right side). The upper panels summarize the BOLD time series in the dorsal hippocampus when there was no electrical stimulation of the perforant pathway, and the lower panels summarize the BOLD time series when the perforant pathway was electrically stimulated (indicated by an arrow). The corresponding averaged BOLD time series are shown in Figure 2A-D of the main manuscript.

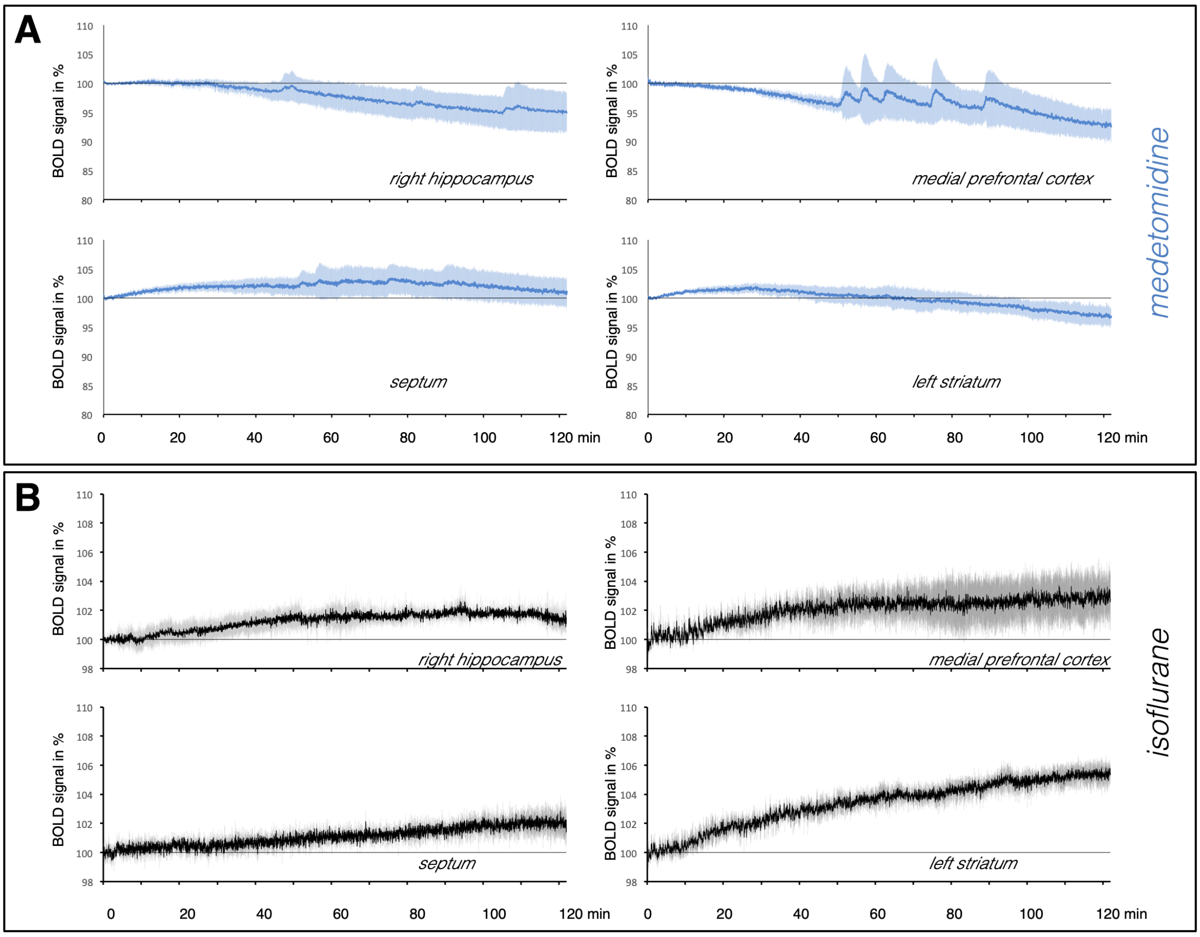

<u>Figure S3.</u>

A BOLD time series from different brain regions during a 2h measurement in presence of medetomidine (without stimulation). Whereas in the hippocampus and prefrontal cortex BOLD signals decline after a certain time, BOLD signals increase in the septum and transiently in the striatum. Note also, that the so called ‘spontaneous BOLD waves’ appeared at different time points in the hippocampus and prefrontal cortex and that they were much stronger in the prefrontal cortex. **B** BOLD time series from different brain regions during a 2h measurement in presence of isoflurane (without stimulation). While BOLD signals increased throughout the experiment in all regions analyzed, the slope varied and was most pronounced in the striatum.

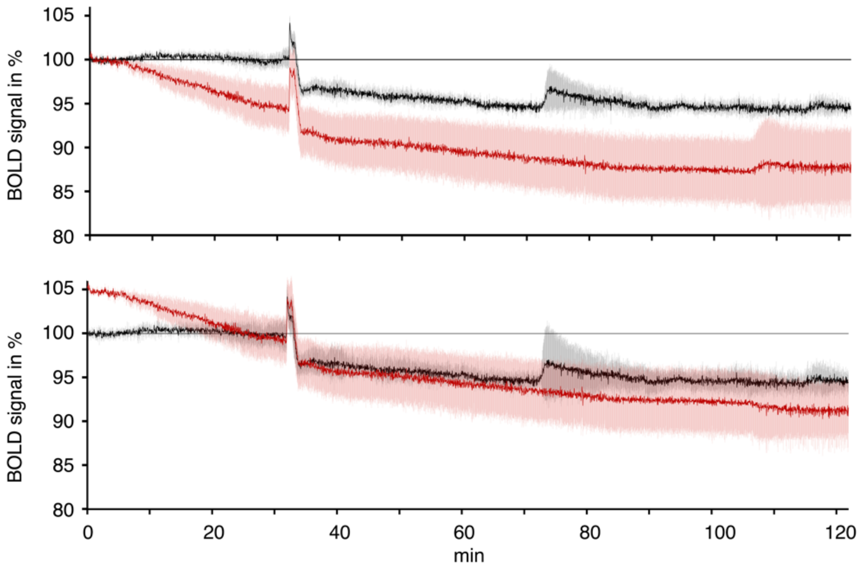

Figure S4.
Stimulation of the right perforant pathway with 20 Hz pulses for 8 seconds caused a similar sustained BOLD signal decline in presence or absence of acetaminophen. (**A**) BOLD time series in the right dorsal hippocampus in a stimulation experiment in absence (black line) or presence of acetaminophen (red line). Acetaminophen caused already a decline of BOLD signals before the stimulation, thus at the time point of stimulation baseline BOLD signals were already different. (**B**) Aligning the same BOLD time series, but considering the time period immediately before the stimulation as 100 % revealed that the subsequently nAD-related BOLD signal decline was similar in presence or absence of acetaminophen. Thus, the mechanisms mediating BOLD signal changes after acetaminophen and after nAD were additive.
